# Multifaceted investigations of PSMB8 provides insights into prognostic prediction and immunological target in thyroid carcinoma

**DOI:** 10.1371/journal.pone.0323013

**Published:** 2025-05-07

**Authors:** Yulou Luo, Yinghui Ye, Yilina Saibaidoula, Yuting Zhang, Yan Chen

**Affiliations:** 1 Department of Breast Surgery, Affiliated Tumor Hospital of Xinjiang Medical University, Urumqi, Xinjiang Uygur Autonomous Region, China; 2 Department of Laboratory Medicine, Xinhua Hospital, Shenzhen, Guangdong Province, China; 3 Department of Breast Surgery, The First Affiliated Hospital, Jinan University, Guangzhou, Guangdong Province, China; 4 Department of Biochemistry and Molecular Biology, School of Basic Medical Sciences, Xinjiang Medical University, Urumqi, Xinjiang Uygur Autonomous Region, China; 5 Xinjiang Key Laboratory of Molecular Biology for Endemic Diseases, Urumqi, Xinjiang Uygur Autonomous Region, China; University of California, Davis, UNITED STATES OF AMERICA

## Abstract

The Proteasome 20S subunit beta 8 (PSMB8) is an integral element of the immunoproteasome complex, playing a pivotal role in antigen processing. Despite its significance, the contributory role of PSMB8 in oncogenesis, particularly in thyroid carcinoma (THCA), has not been well-characterized. To address this gap in knowledge, our study endeavored to delineate the potential associations between PSMB8 and THCA. Transcriptomic profiles and clinical data of patients with THCA were retrieved from The Cancer Genome Atlas (TCGA) database to facilitate comprehensive analysis. Complementary resources from additional online databases were utilized to augment the study. Logistic regression analysis was employed to elucidate the relationship between PSMB8 and various clinicopathological parameters. Uni/multivariate Cox regression analyses were conducted to ascertain the independent prognostic factors for THCA patient outcomes. Quantitative polymerase chain reaction (qPCR) and western blot assays were employed to verify the expression level of PSMB8 *in vitro*. Our study demonstrated that PSMB8 was significantly upregulated in THCA, with its overexpression correlating with lymph node metastasis, extrathyroidal extension, and favorable prognosis. Immunohistochemistry substantiated a higher PSMB8 protein presence in THCA tissue compared to the normal, supporting its potential role as a moderately accurate diagnostic biomarker. Logistic regression analysis identified PSMB8 as a significant indicator of the N1 stage, classical histological subtype, and extrathyroidal extension. Age, T stage, and PSMB8 were further determined as independent prognostic factors for THCA. Functional investigations linked PSMB8 to immune processes, evidenced by its association with increased immune cell infiltration and higher stromal/immune scores, as well as a positive co-expression with several immune checkpoints. A constructed predicted competing endogenous RNA (ceRNA) network implicated PSMB8 in complex post-transcriptional regulation. Finally, *in vitro* assays confirmed the upregulation of PSMB8, underscoring its relevance in THCA and as a target for future research. Our work has preliminarily appraised PSMB8 as a biomarker with certain prognostic and diagnostic significance, and as a potential target for immunotherapy in THCA.

## Introduction

Thyroid carcinoma (THCA) is characterized by a high incidence rate and a prolonged disease course, significantly affecting patient quality of life and imposing a substantial societal health burden. According to global cancer statistics from 2020, THCA was the eleventh most common malignancy worldwide. Among women, its incidence rose to the fifth highest, while its mortality accounted for 0.4% of all cancer-related deaths [1]. THCA includes four main histological subtypes: papillary thyroid carcinoma (PTC), follicular thyroid carcinoma (FTC), anaplastic thyroid carcinoma (ATC), and medullary thyroid carcinoma (MTC). PTC, FTC, and ATC originate from thyroid follicular epithelial cells [2], whereas MTC arises from parafollicular cells, or C cells [3]. PTC and FTC, known as differentiated thyroid carcinoma (DTC), make up over 95% of clinical THCA cases and often initially present as neck lymphadenopathy [3–5]. While DTC generally has a favorable prognosis, ATC is associated with aggressive local invasion and poor survival outcomes [6,7]. MTC, which can secrete thyrocalcitonin, often presents additional symptoms such as flushing and diarrhea [8]. Moreover, more than a quarter of MTC patients have inherited multiple endocrine neoplasia syndrome (MENS) [9]. Despite anatomical similarities, these four subtypes exhibit a wide range of clinical behaviors, from high survival rates in well-differentiated PTC to rapid deterioration in ATC [6,7]. Therefore, it is crucial to explore the pathogenesis and therapeutic strategies for THCA. This includes elucidating the complex molecular mechanisms underlying its onset and progression, examining potential interactions within the cancer-immune microenvironment, and identifying robust biomarkers to improve the accuracy of clinical diagnostics, prognostics, and targeted drug development.

The proteasome 20S is a multicatalytic protease complex composed of α- and β-subunits, playing a crucial role in protein degradation and antigen processing [10]. The constitutive proteasome (CP), an evolutionarily conserved entity in eukaryotes, can undergo subunit replacement in response to immune stimuli. Its active β-subunits (β1, β2, and β5) are replaced by inducible counterparts (β1i, β2i, and β5i), forming the immunoproteasome (IP) complex [11]. This structural change enhances the IP’s ability to generate MHC class I-associated peptides, improving both their quality and quantity [12–15], and increasing their antigenicity to CD8^+^ T cells [16]. The inducible catalytic subunit β5i, also known as low molecular mass peptide 7 (LMP7) or proteasome 20S subunit beta 8 (PSMB8), is encoded by the *PSMB8* gene located on human chromosome 6 (p21.32) [17,18]. PSMB8 is prevalent in the cytoplasm, nucleus, and extracellular space [19] and was initially linked to proteasome-related auto-inflammatory syndromes [17,20–22]. Recent studies have implicated PSMB8 in cancer progression, where it acts as a regulatory molecule [23–26]. Its upregulation is a common genetic alteration in various cancer types, often associated with poor prognoses [27,28], although contrasting trends have been observed in triple-negative breast cancer and lung cancer [29,30]. Beyond prognostication, PSMB8 has been validated as a biomarker for responsiveness to immunotherapy and radiotherapy; patients with elevated PSMB8 expression show increased sensitivity to these treatments [31,32]. High PSMB8 levels also correlate with more active immune pathways, a greater presence of antitumor immune cells, fewer protumor immune cells, and higher immunoscores [30], underscoring its significant role in tumor immunology. Additionally, PSMB8 may serve as a target for non-coding RNAs, such as miR-451a, during carcinogenesis [25,33,34]. However, the detailed oncogenic mechanisms of PSMB8 and its interactions with tumor immunology and non-coding RNAs remain unclear. Importantly, the full potential of PSMB8 as a prognostic/diagnostic and immunological biomarker in THCA has yet to be fully explored.

In this study, we primarily explored the associations between PSMB8 and THCA using bioinformatics, aiming to enrich the existing literature and provide a foundation for further elucidation and experimental validation. We conducted comprehensive investigations into the diagnostic and prognostic value of PSMB8, as well as its correlation with immune cells and genes in THCA. Additionally, we established a competing endogenous RNA (ceRNA) network. The expression level of PSMB8 in THCA was verified through quantitative polymerase chain reaction (qPCR) and western blot assays.

## Materials and methods

### Raw data acquisition and preprocessing

We obtained and preprocessed clinical information and RNA-sequencing data of THCA from The Cancer Genome Atlas (TCGA) (https://www.cancer.gov), which included 510 THCA samples (papillary thyroid carcinoma) and 58 normal samples. RNA-sequencing profiles across 33 cancer types were also acquired from TCGA for pan-cancer analysis, encompassing 10,363 tumor samples and 730 normal samples. We then categorized the 510 THCA samples into high and low PSMB8 expression subgroups based on the median PSMB8 expression value. Since the personal information of samples from TCGA was anonymized, thus ethnics approval and consent to participate were not needed.

Approximately 3.5% of the clinical information in the THCA samples had missing data, primarily in secondary clinical information such as residual tumor, thyroid gland disorder history, and disease-free interval (DFI). Since these missing data were not expected to significantly impact the main analysis results, they were excluded from analyses involving these aspects. For the transcriptome data, box plots and Z-scores were used to identify outliers, which were removed to prevent them from affecting subsequent analysis results.

### Gene expression analysis

We utilized ggplot2 R package for expression analysis, examining the expression pattern of PSMB8 across 33 cancer types and its association with various clinicopathological characteristics in the TCGA-THCA cohort. Diagnostic receiver operating characteristic (ROC) curves were generated using the pROC and ggplot2 R packages. Immunohistochemistry (IHC) results for PSMB8 protein in both THCA and normal tissues were obtained from The Human Protein Atlas (HPA) (http://www.proteinatlas.org). Additionally, we compared the baseline characteristics of THCA patients between high and low PSMB8 expression subgroups and conducted logistic regression analysis to identify PSMB8-associated risk factors.

### Survival analysis

We employed the survminer and survival R packages to assess overall survival (OS), disease-specific survival (DSS), DFI, and progression-free interval (PFI) between high and low PSMB8 expression subgroups in THCA patients. We also evaluated survival probabilities in various clinical subgroups stratified by PSMB8 expression levels. Independent prognostic factors were identified through univariate and multivariate Cox regression analyses. Using these factors, we constructed a nomogram with the rms and survival R packages to predict the survival probability of THCA patients. To quantify the predictive accuracy of the nomogram over time, we calculated the area under the curve (AUC) for time-dependent ROC curves from 1 to 14 years using the timeROC and ggplot2 R packages.

### Functional characterization

We extracted a PSMB8-centric protein-protein interaction (PPI) network from the STRING database (http://cn.string-db.org) [35]. To identify differentially expressed genes (DEGs) between high and low PSMB8 expression subgroups, we used the DESeq2 R package to generate a volcano plot, with the low PSMB8 expression subgroup serving as the reference [36]. Genes with an adjusted *P* value ≤ 0.05 and | Log_2_FoldChange| > 1 were considered differentially expressed. Subsequently, all identified DEGs were analyzed using Gene Set Enrichment Analysis (GSEA) via the clusterProfiler R package [37,38]. Additionally, the top 50 upregulated DEGs (Log_2_FoldChange > 1) in the high PSMB8 expression subgroup were subjected to Gene Ontology (GO) and Kyoto Encyclopedia of Genes and Genomes (KEGG) functional enrichment analyses using the clusterProfiler and org.Hs.e.g.,db R packages [37].

### Immune-related analysis

The infiltration levels of 24 immune cells were quantified using the ssGSEA algorithm in GSVA R package [39,40]. We then assessed the significance and correlation between PSMB8 expression and the infiltration levels of these immune cells. The stromal and immune scores were quantified using the ESTIMATE algorithm in estimate R package [41]. We evaluated the significance and correlation between PSMB8 expression and these scores. Furthermore, the significance and correlation between PSMB8 expression and 47 immune checkpoints were illustrated using the ggplot2 R package. These immune checkpoints were retrieved from a previous study [42]. Additionally, we explored the significance and correlation between PSMB8 expression and four immune-related gene clusters: chemokine genes, chemokine receptor genes, immune activation genes, and immune suppression genes, through co-expression analysis. Each cluster includes genes with similar functions in the immune response, as defined in a prior study [43]. After excluding genes with *P* > 0.05, we performed Least Absolute Shrinkage and Selection Operator (LASSO) regression analysis to identify potential prognostic risk factors from these gene clusters.

### ceRNA network construction

starBase (http://starbase.sysu.edu.cn) was employed to predict microRNAs (miRNAs) that might target PSMB8 mRNA. miRNA with significantly negative correlation with PSMB8 in THCA was selected as candidate (|*r*| > 0.1, *P* ≤ 0.05). We identified the potential binding sites between PSMB8 mRNA and the predicted miRNAs using TargetScan (http://targetscan.org). starBase and miRNet (http://mirnet.ca/miRNet) were subsequently utilized to predict upstream long non-coding RNAs (lncRNAs) that may target the predicted miRNAs, and the results from both databases were overlapped. The correlation and significance between the lncRNAs and the predicted miRNAs were determined using starBase. lncRNA with significantly negative correlation with the predicted miRNA in THCA was selected as candidate (|*r*| > 0.1, *P* ≤ 0.05). Finally, the competing endogenous RNA (ceRNA) network was constructed using the ggalluvial R package.

### Cell culture

Human THCA cell lines, KTC-1 (No. CL-0649) and B-CPAP (No. CL-0575), and human normal thyroid epithelial cell line, Nthy-ori 3–1 (No. CL-0817) were purchased from Wuhan Procell Life Science and Technology Co., Ltd. (Wuhan, China). All the three cell lines have been authenticated by short tandem repeat (STR) profiling according to the supplier. Each cell line was cultured in its dedicated medium (Wuhan Procell Life Science and Technology Co. Ltd., Wuhan, China). Cells were cultured in RPMI-1640 (Gibco-BRL), supplemented with 10% foetal bovine serum (Bioserum), 100 U/mL penicillin G and 100 μg/mL streptomycin. Cells were grown at 37°C in a 90% humidified atmosphere of 5% CO_2_ and maintained in the tissue culture incubator.

### Quantitative PCR

Total RNA was extracted from THCA cell lines and Nthy-ori 3–1 using TRIzol Reagent (No. P118-05, GenStar, Beijing, China) according to the manufacturer’s instructions. Total RNA was amplified by qPCR using SYBR Green Master Mix (No. C0006, TOPSCIENCE, Shanghai, China) according to the manufacturer’s instructions. The relative expression of PSMB8 was determined by the 2^−ΔΔCt^ calculation formula in contrast to GAPDH expression. The reason for selecting GAPDH as internal control was supported by its well-documented stability and consistent expression levels across various types of thyroid cancer cell lines, as reported in previous studies [44–47]. Furthermore, GAPDH is commonly used in similar bioinformatics analyses and has been validated as a reliable internal control under various experimental conditions [48–50]. The primer pairs of PSMB8 and GAPDH were synthesized by Accurate Biology (Changsha, China). PSMB8 Forward: 5’-CACGCTCGCCTTCAAGTTC-3’ and Reverse: 5’-AGGCACTAATGTAGGACCCAG-3’; GAPDH Forward: 5’-GTCTCCTCTGACTTCAACAGCG-3’ and Reverse: 5’-ACCACCCTGTTGCTGTAGCCAA-3’.

### Western blot

THCA cells and normal thyroid epithelial cells were harvested with radioimmuno precipitation assay (RIPA) buffer (Zhonghuihecai, Xi’an, China) and pelleted by centrifugation at 4°C for 15 min, and the supernatant was discarded. Next, 1/5 sodium dodecyl sulfate-polyacrylamide gel (SDS-PAGE) sample loading buffer, 5× (Beyotime, Shanghai, China) was added to the supernatant and heated in a 100°C metal bath for 10 min. The protein was separated on a 15% SDS-PAGE, transferred to a 0.22-mm polyvinylidene fluoride (PVDF) membrane (Millipore, USA), placed in 5% skimmed milk, blocked for approximately 2h, and incubated with specific antibodies. Blots were then incubated with horseradish peroxidase (Goat, No.C31430100, No. C31460100, 1:2000, Invitrogen). Specific antibodies used were as follows: PSMB8 (Rabbit, No. PA1–972, 1:2,000, Invitrogen) and β-actin (Mouse, No. Ab6276, 1:100,000, Abcam). The protein bands were enhanced using a chemiluminescent kit (Vazyme, Nanjing, China). All images were captured by the Bio-Rad Imaging System.

### Statistical analysis

Statistical analysis and bioinformatic analysis were all conducted by R 4.0.3. The expressive correlation was identified by Spearson’s R and statistical significance. | *r*| > 0.1 was considered to be relevant and *P* ≤ 0.05 was deemed as statistically significant. Chi-square test (χ^2^) and normal approximation method of the non-parametric rank sum test (z) were used for the comparison of clinicopathological characteristics between subgroups. Log-rank test was employed for survival analysis. * indicates *P* ≤ 0.05, ** indicates *P* ≤ 0.01 and *** indicates *P* ≤ 0.001 throughout this study.

## Results

### Transcriptic level of PSMB8 expression and IHC results

**Fig 1:**
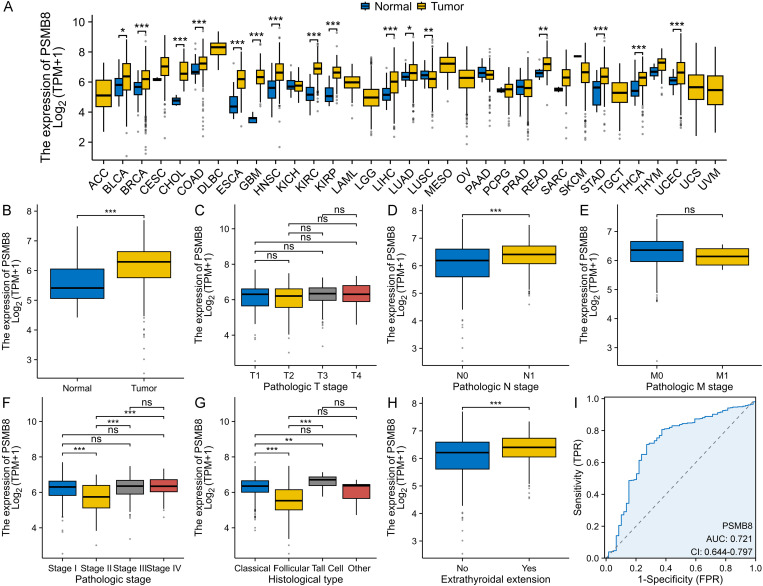
Transcriptic expression pattern and diagnostic value of PSMB8. **(A)** PSMB8 was differentially expressed across 16 cancer types. **(B)** PSMB8 expression was approximately 14.8% higher than that in normal samples. **(C)** No significant differential expression of PSMB8 was observed within the T stage subgroups. **(D)** PSMB8 expression was significantly upregulated in patients with lymphnode metastasis. **(E)** No significant differential expression of PSMB8 was observed within the M stage subgroups. **(F)** The expression level of PSMB8 in pathological stage II was notably lower in comparison to the other three stages **(G)** The follicular subtype exhibited the lowest PSMB8 expression levels compared to the other subtypes. **(H)** PSMB8 expression was significantly upregulated in patients with extrathyroidal extension. **(I)** The diagnostic value of PSMB8 for distinguishing THCA from the normal was supported by an AUC value of 0.721. PSMB8, proteasome 20S subunit beta 8; TPM, transcripts per million; ACC, adrenocortical cancer; BLCA, bladder cancer; BRCA, breast cancer; CESC, cervical cancer; CHOL, bile duct cancer; COAD, colon cancer; DLBC, diffuse large B-cell lymphoma; ESCA, esophageal cancer; GBM, glioblastoma; HNSC, head and neck cancer; KICH, kidney chromophobe; KIRC, kidney clear cell carcinoma; KIRP, kidney papillary cell carcinoma; LAML, acute myeloid leukemia; LGG, lower grade glioma; LIHC, liver hepatocellular cancer; LUAD, lung adenocarcinoma; LUSC, lung squamous cell carcinoma; MESO, mesothelioma; OV, ovarian cancer; PAAD, pancreatic cancer; PCPG, pheochromocytoma & paraganglioma; PRAD, prostate cancer; READ, rectal cancer; SARC, sarcoma; SKCM, melanoma; STAD, stomach cancer; TGCT, testicular cancer; THCA, thyroid carcinoma; THYM, thymoma; UCEC, endometrioid cancer; UCS, uterine carcinosarcoma; UVM, ocular melanomas; AUC, area under the curve; CI, confidence interval; FPR, false positive rate; TPR, true positive rate; ROC, receiver operating characteristic.

**Fig 2:**
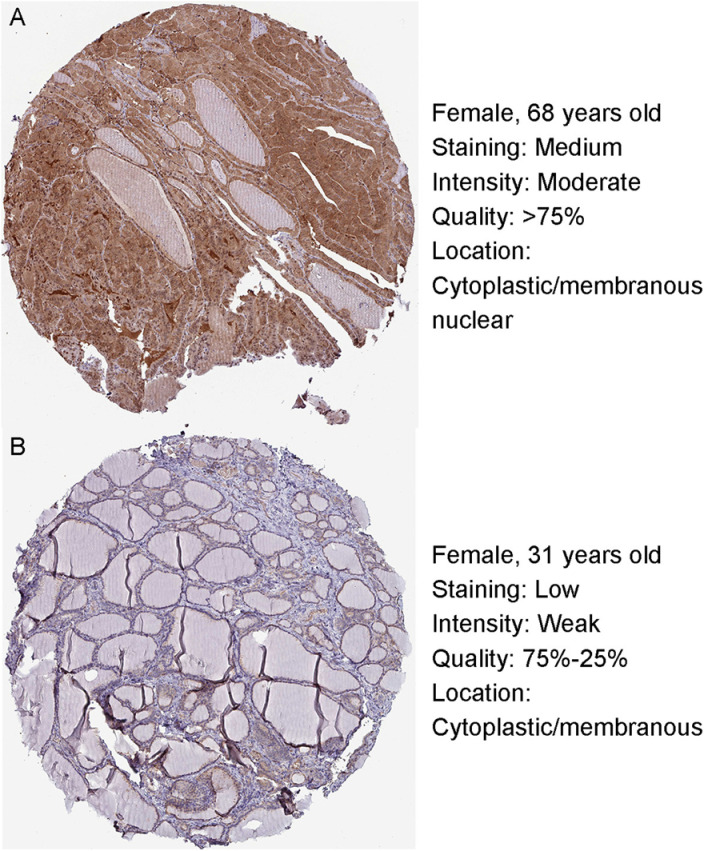
Verification of PSMB8 upregulation in THCA by IHC staining. **(A)** IHC result of PSMB8 protein in THCA tissue. Ultra link: https://www.proteinatlas.org/ENSG00000204264-PSMB8/pathology/thyroid+cancer#img. **(B)** IHC result of PSMB8 protein in normal thyroid epithelial tissue. Ultra link: https://www.proteinatlas.org/ENSG00000204264-PSMB8/tissue/thyroid+gland#img. IHC, immunohistochemistry; PSMB8, proteasome 20S subunit beta 8; THCA, thyroid carcinoma.

We observed a significant differential expression of PSMB8 between tumor and normal samples across 16 cancer types (Fig 1A). In THCA samples specifically, PSMB8 expression was approximately 14.8% higher than in normal samples (Fig 1B). No significant differential expression of PSMB8 was detected within the T stage or M stage subgroups (Fig 1C and 1E). However, PSMB8 expression was significantly upregulated in patients with lymph node metastasis and extrathyroidal extension (Fig 1D and 1H). Additionally, PSMB8 expression in pathological stage II was notably lower compared to the other three stages (Fig 1F). Among the histological subtypes of THCA, the follicular subtype exhibited the lowest PSMB8 expression levels (Fig 1G). The diagnostic accuracy of PSMB8 for distinguishing THCA from normal tissue was supported by an AUC value of 0.721 in the ROC curve (Fig 1I). IHC results further confirmed the upregulation of PSMB8 protein in THCA tissue compared to normal tissue (Fig 2A and 2B).

### Clinical correlation and survival analysis

Patients with high PSMB8 expression showed a higher incidence of lymph node metastasis (χ^2^ = 10.65, *P* < 0.005) and extrathyroidal extension (χ^2^ = 8.51, *P* < 0.005) (Table 1). Logistic regression analysis identified N1 stage (OR = 1.842, 95% CI: 1.273–2.665, *P* = 0.001), classical histological subtype (OR = 2.016, 95% CI: 1.361–2.986, *P* < 0.001), and extrathyroidal extension (OR = 1.866, 95% CI: 1.269–2.758, *P* = 0.002) as risk factors associated with PSMB8 (Table 2).

**Table 1:** Association between PSMB8 expression and clinicopathological characteristics.

Clinicopathological characteristics	Low expression of PSMB8	High expression of PSMB8	Statistics	*P*-value
Age	255	255	0.39 (χ^2^)	*P* > 0.05
≤45	117 (45.88%)	124 (48.63%)		
>45	138 (54.12%)	131 (51.37%)		
Race	195	219	0.001 (z)	*P* > 0.05
Asian	20 (10.26%)	30 (14.16%)		
Black	13 (6.67%)	14 (6.39%)		
White	162 (83.08%)	174 (79.45%)		
American Indian	0	1 (0.46%)		
Gender	255	255	0.01 (χ^2^)	*P* > 0.05
Female	185 (72.55%)	186 (72.94%)		
Male	70 (27.45%)	69 (27.06%)		
T stage	254	254	0.006 (z)	*P* > 0.05
T1	69 (27.17%)	74 (29.13%)		
T2	93 (36.61%)	74 (29.13%)		
T3	81 (31.89%)	94 (37.01%)		
T4	11 (4.33%)	12 (4.72%)		
N stage	218	242	10.65 (χ^2^)	*P* < 0.005
N0	126 (57.80%)	103 (42.56%)		
N1	92 (42.20%)	139 (57.44%)		
M stage	133	162	1.75 (χ^2^)	*P* > 0.05
M0	127 (95.49%)	159 (98.15%)		
M1	6 (4.51%)	3 (1.85%)		
Pathological stage	254	254	0.03 (z)	*P* > 0.05
Stage I	139 (54.72%)	147 (57.87%)		
Stage II	38 (14.96%)	14 (5.51%)		
Stage III	54 (21.26%)	59 (23.23%)		
Stage IV	23 (9.06%)	34 (13.39%)		
Histological type	255	255	0.002 (z)	*P* > 0.05
Classical	164 (64.31%)	200 (78.43%)		
Follicular	81 (31.76%)	20 (7.84%)		
Tall cell	6 (2.35%)	30 (11.76%)		
Other	4 (1.57%)	5 (1.96%)		
Tumor status	245	253	2.24 (χ^2^)	*P* > 0.05
With tumor	28 (11.43%)	19 (7.51%)		
Tumor free	217 (88.57%)	234 (92.49%)		
Residual tumor	221	227	0.002 (z)	*P* > 0.05
R0	197 (89.14%)	193 (85.02%)		
R1	23 (10.41%)	31 (13.66%)		
R2	1 (0.45%)	3 (1.32%)		
Extrathyroidal extension	246	246	8.51 (χ^2^)	*P* < 0.005
No	184 (74.80%)	154 (62.60%)		
Yes	62 (25.20%)	92 (37.40%)		
Thyroid gland disorder history	229	223	0.04 (z)	*P* > 0.05
Lymphocytic thyroiditis	29 (12.66%)	45 (20.18%)		
Nodular hyperplasia	44 (19.21%)	24 (10.76%)		
Normal	144 (62.88%)	141 (63.23%)		
Other	12 (5.24%)	13 (5.83%)		

PSMB8, proteasome 20S subunit beta 8.

**Table 2:** Logistic regression analysis associated with PSMB8 in THCA.

Clinicopathological characteristics	Total (N)	Odds ratio (OR)	*P*-value
T stage (T4&T3 vs. T2&T1)	510 (196 vs. 314)	1.260 (0.882-1.801)	0.204
N stage (N1 vs. N0)	462 (232 vs. 230)	1.842 (1.273-2.665)	0.001
M stage (M1 vs. M0)	295 (9 vs. 286)	0.399 (0.098-1.628)	0.200
Pathological stage (Stage IV&III vs. Stage II&I)	510 (171 vs. 339)	1.327 (0.917-1.920)	0.133
Histological type (Classical vs. Follicular&Other&Tall Cell)	510 (365 vs. 145)	2.016 (1.361-2.986)	< 0.001
Extrathyroidal extension (Yes vs. No)	492 (154 vs. 338)	1.866 (1.269-2.758)	0.002
Age (> 45 vs. ≤ 45)	510 (271 vs. 239)	0.896 (0.633-1.268)	0.536
Gender (Male vs. Female)	510 (140 vs. 370)	0.942 (0.638-1.391)	0.766

PSMB8, proteasome 20S subunit beta 8; THCA, thyroid carcinoma.

**Fig 3:**
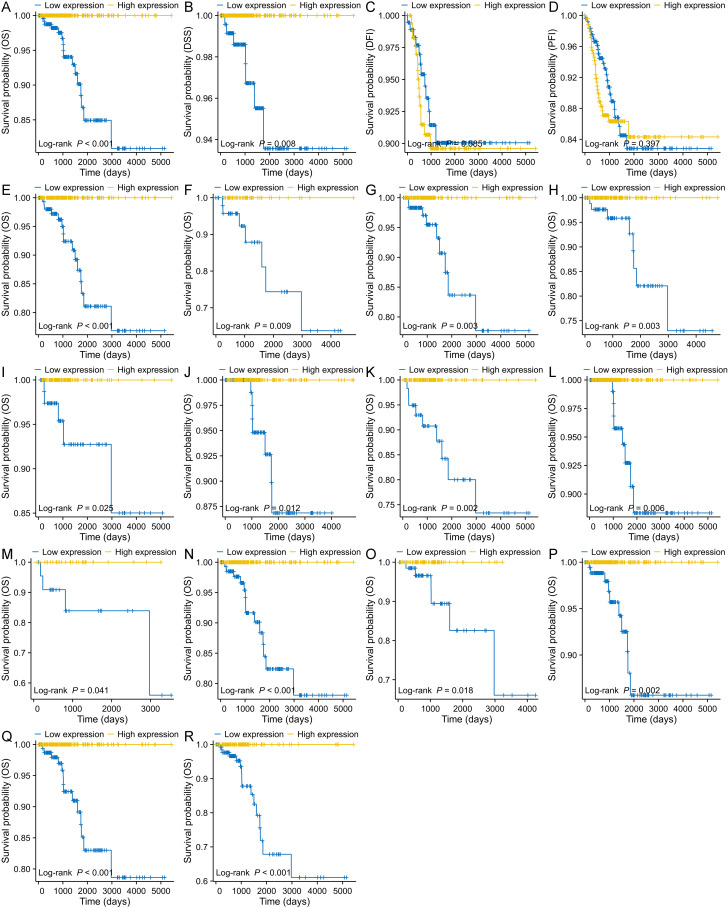
Survival analysis of PSMB8 in THCA. **(A)** High PSMB8 expression inferred significantly better OS for patients with THCA. **(B)** High PSMB8 expression inferred significantly better DSS for patients with THCA. **(C)** PSMB8 expression was not correlated with DFI for patients with THCA. **(D)** PSMB8 expression was not correlated with PFI for patients with THCA. High PSMB8 expression inferred significantly better OS in various clinical subgroups: (E) classical histological subtype, (F) pathological stage III, **(G)** M0 stage, **(H)** N1 stage, **(I)** T3 stage, (J) extrathyroidal extension (Yes), (K) extrathyroidal extension (No), (L) residual tumor R0, (M) residual tumor R1, (N) patients without a history of thyroid gland disorders, (O) male, (P) female, (Q) white race, and (R) age > 45). OS, overall survival; DSS, disease specific survival; DFI, disease free interval; PFI, progression free interval; HR, hazard ratio; PSMB8, proteasome 20S subunit beta 8; THCA, thyroid carcinoma.

**Fig 4:**
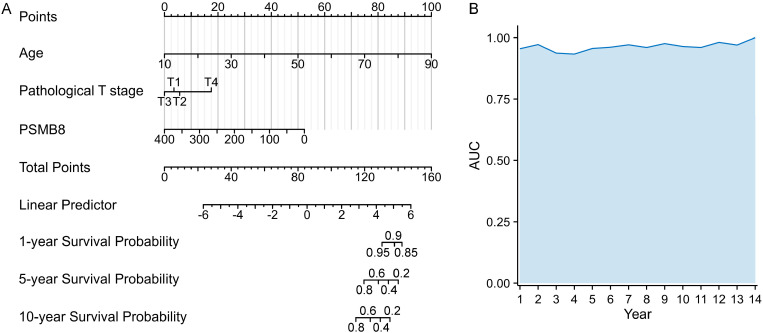
Construction of the nomogram to predict survival probability in THCA. **(A)** The nomogram containing three variables (age, T stage, and PSMB8 expression) was constructed. **(B)** The predictive capability of the nomogram was excellent supporting by high AUCs. PSMB8, proteasome 20S subunit beta 8; AUC, area under the curve; THCA, thyroid carcinoma; ROC, receiver operating characteristic.

Survival analysis revealed that THCA patients with high PSMB8 expression had significantly better OS and DSS, with these favorable outcomes persisting beyond 4000 days (Fig 3A and 3B). However, high PSMB8 expression did not correlate with DFI or PFI (Fig 3C and 3D). Subgroup analyses showed a significant association between high PSMB8 expression and improved OS in various clinical subgroups, including the classical histological subtype (Fig 3E), pathological stage III (Fig 3F), M0 stage (Fig 3G), N1 stage (Fig 3H), T3 stage (Fig 3I), with extrathyroidal extension (Fig 3J), without extrathyroidal extension (Fig 3K), residual tumor R0 (Fig 3L), residual tumor R1 (Fig 3M), patients without a history of thyroid gland disorders (Fig 3N), male (Fig 3O), female (Fig 3P), white race (Fig 3Q), and age > 45 (Fig 3R). Cox regression analyses identified age (HR = 1.131, 95% CI: 1.080–1.183, *P* < 0.001), T3 stage (HR = 0.229, 95% CI: 0.060–0.881, *P* < 0.05), and PSMB8 expression (HR = 0.987, 95% CI: 0.974–1.000, *P* < 0.05) as independent prognostic factors for THCA (Table 3). A novel nomogram incorporating age, T stage, and PSMB8 expression was constructed to predict the survival probability for THCA patients (Fig 4A), demonstrating strong predictive accuracy with a concordance index (C-index) of 0.947 (95% CI: 0.933–0.961). The nomogram’s predictive capability was further validated by calculating the AUC for time-dependent ROC curves, ranging from 1 to 14 years (Fig 4B).

**Table 3:** Uni/multivatiate Cox regression analysis of clinicopathological characteristics and PSMB8 expression.

Clinicopathological characteristics	Total (N)	Univariate analysis		Multivariate analysis	
		HR (95% CI)	*P*-value	HR (95% CI)	*P*-value
Age	510	1.120 (1.078-1.164)	< 0.001	1.131 (1.080-1.183)	< 0.001
Gender	510		0.193		
Female	371	Reference			
Male	139	1.963 (0.710-5.428)	0.193		
Pathological T stage	508		<0.001		
T4	23	Reference			
T2	167	0.089 (0.022-0.359)	<0.001	0.416 (0.090-1.917)	0.260
T1	143	0.087 (0.017-0.434)	0.003	0.335 (0.059-1.907)	0.218
T3	175	0.139 (0.042-0.458)	0.001	0.229 (0.060-0.881)	0.032
Pathological N stage	510		0.548		
N1	231	Reference			
N0	229	0.693 (0.226-2.123)	0.521		
NX	50	1.539 (0.407-5.818)	0.525		
Pathological M stage	509		0.083		
M0	286	Reference			
Mx	214	0.691 (0.230-2.074)	0.510	1.053 (0.303-3.655)	0.935
M1	9	4.419 (0.948-20.605)	0.059	3.193 (0.544-18.742)	0.199
PSMB8	510	0.985 (0.974-0.996)	0.006	0.987 (0.974-1.000)	0.043

PSMB8, proteasome 20S subunit beta 8; HR, hazard ratio; CI, confidence interval.

### Functional characterization

**Fig 5:**
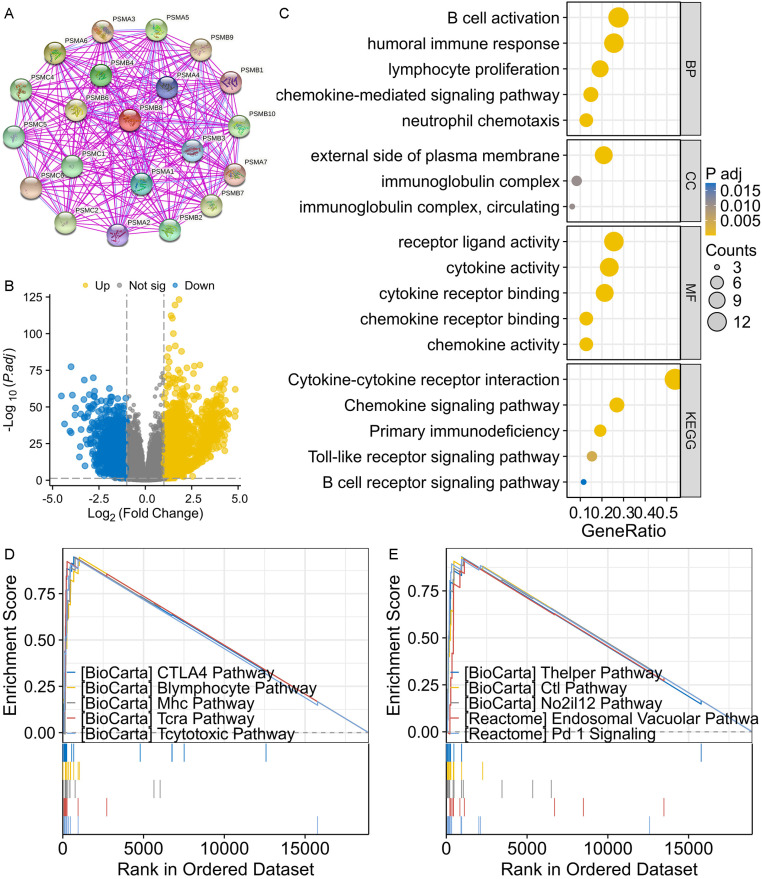
Functional enrichment analysis of PSMB8 in THCA. **(A)** PSMB8-centered PPI network. **(B)** Volcano plot showed that 667 downregulated DEGs and 752 upregulated DEGs were identified between low/high PSMB8 expression subgroups. **(C)** GO/KEGG functional enrichment analysis implied that PSMB8 was associated with multiple immune-related signaling pathways and biological processes, especially relevant to cytokine and chemokine. **(D-E)** GSEA functional enrichment analysis suggested that PSMB8 was associated with multiple immune-related signaling pathways. PSMA1, proteasome 20S subunit alpha 1; PSMA2, proteasome 20S subunit alpha 2; PSMA3, proteasome 20S subunit alpha 3; PSMA4, proteasome 20S subunit alpha 4; PSMA5, proteasome 20S subunit alpha 5; PSMA6, proteasome 20S subunit alpha 6; PSMA7, proteasome 20S subunit alpha 7; PSMB1, proteasome 20S subunit beta 1; PSMB2, proteasome 20S subunit beta 2; PSMB3, proteasome 20S subunit beta 3; PSMB4, proteasome 20S subunit beta 4; PSMB6, proteasome 20S subunit beta 6; PSMB7, proteasome 20S subunit beta 7; PSMB8, proteasome 20S subunit beta 8; PSMB9, proteasome 20S subunit beta 9; PSMB10, proteasome 20S subunit beta 10; PSMC1, proteasome 26S subunit, ATPase 1; PSMC2, proteasome 26S subunit, ATPase2; PSMC4, proteasome 26S subunit, ATPase4; PSMC5, proteasome 26S subunit, ATPase5; PSMC6, proteasome 26S subunit, ATPase6; THCA, thyroid carcinoma; PPI, protein-protein network; DEGs, differentially expressed genes; GO, Gene Ontology; KEGG, Kyoto Encyclopedia of Genes and Genomes; GSEA, Gene Set Enrichment Analysis.

The PPI network centered on PSMB8 indicated that the key interactors were primarily proteasome subunits (Fig 5A). A total of 1,419 genes were identified as DEGs, with 667 being downregulated and 752 upregulated (Fig 5B). GO/KEGG functional enrichment analysis revealed that DEGs upregulated in the high PSMB8 expression subgroup were predominantly involved in processes such as humoral immune response, B cell activation, lymphocyte proliferation, and localized to the external side of the plasma membrane. They were also significant in the immunoglobulin complex, receptor ligand activity, cytokine activity, cytokine-cytokine receptor interaction, and chemokine signaling pathway, among others (Fig 5C). GSEA highlighted the top 10 statistically significant terms, with the CTLA4 pathway being the most enriched functional annotation related to PSMB8 (Fig 5D and 5E). Other notable PSMB8-associated functional annotations were predominantly related to the immune response, including pathways such as the B lymphocyte, MHC, TCRA, T cytotoxic, T helper, CTL, and PD1 pathways.

### Immune-related analysis

**Fig 6:**
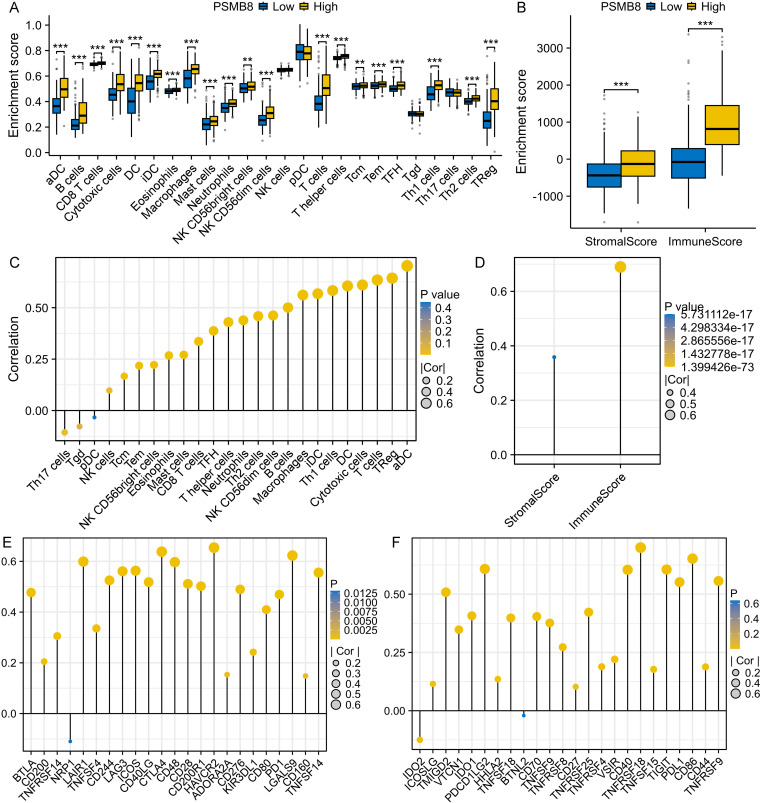
Correlation of PSMB8 expression with immune cells, stromal/immune score and immune checkpoints in THCA. (A) 22 immune cells were significantly more abundant in high PSMB8 expression subgroup. **(B)** Stromal score and immune score were significantly higher in high PSMB8 expression subgroup. (C) 22 immune cells were significantly positively correlated with PSMB8 expression. **(D)** Stromal score and immune score were significantly positively correlated with PSMB8 expression. (E-F) 43 immune checkpoints were significantly positively correlated with PSMB8 expression. PSMB8, proteasome 20S subunit beta 8; THCA, thyroid carcinoma.

We have primarily highlighted the potential association between PSMB8 and immune-related pathways through functional enrichment analysis. Subsequently, we sought to delve deeper into the potential connections between PSMB8, the tumor immune microenvironment, and immune-related genes. Initially, we compared the infiltration levels of 24 immune cells between the high and low PSMB8 expression subgroups (Fig 6A). The high PSMB8 expression subgroup exhibited more abundant infiltration levels of 20 immune cells, including aDC, B cells, CD8 T cells, cytotoxic cells, and mast cells, among others. Additionally, higher stromal scores and immune scores were observed in the high PSMB8 expression subgroup (Fig 6B). A majority of the immune cells showed a significant positive correlation with PSMB8 expression, except for Th17 cells, pDC, and Tgd cells (Fig 6C). aDC emerged as the immune cell most strongly correlated with PSMB8 expression in THCA (*r* = 0.725, *P* < 0.001). Similarly, PSMB8 expression also demonstrated significant positive correlations with both stromal score (*r* = 0.358, *P* < 0.001) and immune score (*r* = 0.690, *P* < 0.001) (Fig 6D). Further analysis revealed that a total of 43 immune checkpoints, including CTLA4, LAG3, PD1, PDL1, and TIGIT, were significantly positively correlated with PSMB8 expression, with correlation coefficients ranging from 0.46 to 0.64 (Fig 6E and 6F).

**Fig 7:**
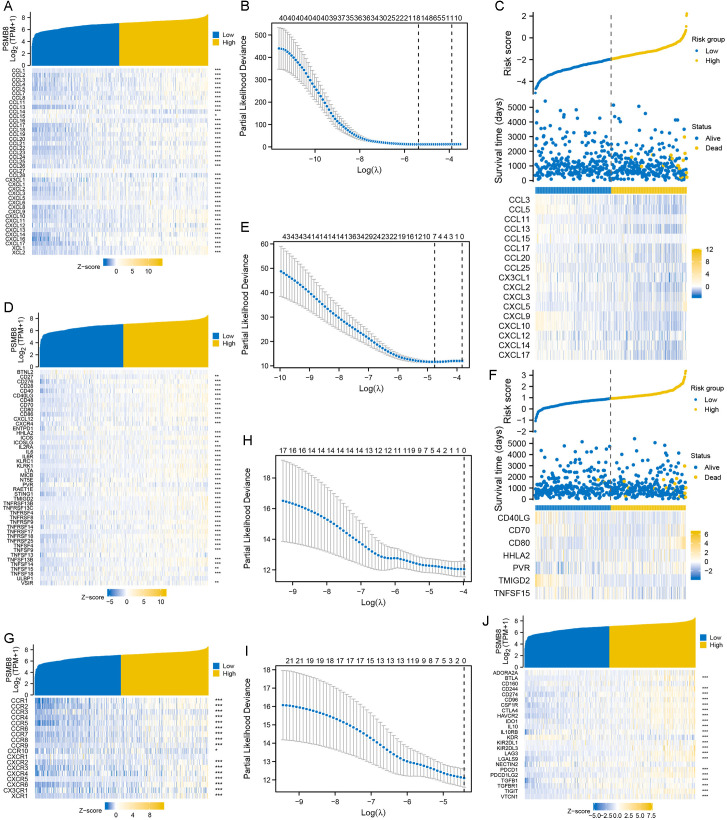
Correlation of PSMB8 expression with four immune-related gene clusters in THCA. **(A)** A majority of chemokine genes were significantly positively correlated with PSMB8 expression. (B) 17 prognostic risk genes were screened by LASSO. **(C)** Expression pattern of the 17 prognostic risk genes between risk groups. **(D)** A majority of immune activation genes were significantly positively correlated with PSMB8 expression. **(E)** Seven prognostic risk genes were screened by LASSO. **(F)** Expression pattern of the seven prognostic risk genes between risk groups. **(G)** A majority of chemokine receptor genes were significantly positively correlated with PSMB8 expression. **(H)** No prognostic risk genes were screened from chemokine receptor genes by LASSO. **(I)** No prognostic risk genes were screened from immune suppression genes by LASSO. **(J)** A majority of immune suppression genes were significantly positively correlated with PSMB8 expression. PSMB8, proteasome 20S subunit beta 8; TPM, transcripts per million; THCA, thyroid carcinoma; LASSO, least absolute shrinkage and selection operator.

To further explore prognostic risk genes associated with PSMB8, we conducted co-expression analyses between PSMB8 and four immune-related gene clusters. The majority of chemokine genes demonstrated a significant positive correlation with high PSMB8 expression (Fig 7A). Subsequent LASSO regression analysis identified 17 prognostic risk genes associated with this cluster (Fig 7B and 7C). Similarly, most immune activation genes were significantly positively correlated with high PSMB8 expression (Fig 7D), with LASSO regression pinpointing seven prognostic risk genes (Fig 7E and 7F). In contrast, while a majority of chemokine receptor genes and immune suppression genes also showed significant positive correlations with high PSMB8 expression (Fig 7G and 7J), LASSO regression analysis did not identify any prognostic risk genes from these clusters (Fig 7H and 7I).

### ceRNA network construction

**Fig 8:**
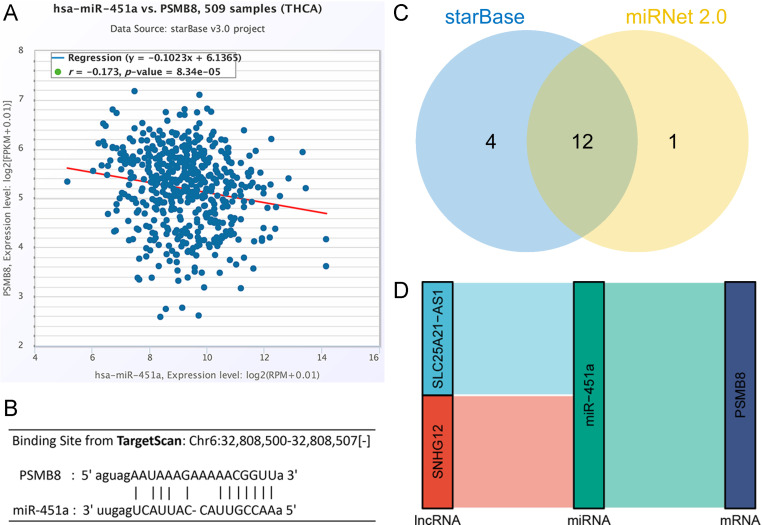
Construction of a PSMB8-related ceRNA network. **(A)** PSMB8 was significantly negatively correlated with miR-451a in THCA. **(B)** The potential binding site between PSMB8 and miR-451a. (C) 12 lncRNAs were overlapped between starBase and miRNet. (D) lncRNA-miR-451a-PSMB8 regulatory axes. PSMB8, proteasome 20S subunit beta 8; THCA, thyroid carcinoma; FPKM, fragments per kilobase of transcript per million fragments mapped; RPM, reads per million mapped reads; SNHG12, small nucleolar RNA host gene 12; SLC25A21-AS1, SLC25A21 antisense RNA 1; ceRNA, competing endogenous RNA.

The lncRNA-miRNA-mRNA axis is a crucial regulatory mechanism within cellular signal transduction pathways. Investigating the ceRNA network provides valuable insights into the complex regulatory landscape surrounding PSMB8 in THCA. Through correlation analysis using starBase, miR-451a was identified as a candidate upstream miRNA (*r* = -0.173, *P* < 0.001) (Fig 8A). The predicted binding site between PSMB8 mRNA and miR-451a was obtained from TargetScan (Fig 8B). A total of 16 lncRNAs were predicted to target miR-451a by starBase, and 13 by miRNet, with 12 lncRNAs commonly predicted by both platforms (Fig 8C). Further correlation analysis from starBase identified two lncRNAs, SNHG12 (*r* = -0.118, *P* = 7.84E-03) and SLC25A21-AS1 (*r* = -0.101, *P* = 2.24E-02), that were significantly negatively correlated with miR-451a in THCA. Ultimately, the predicted ceRNA network derived from PSMB8 in THCA was constructed to illustrate the potential regulatory mechanism (Fig 8D).

### PSMB8 was upregulated in THCA

**Fig 9:**
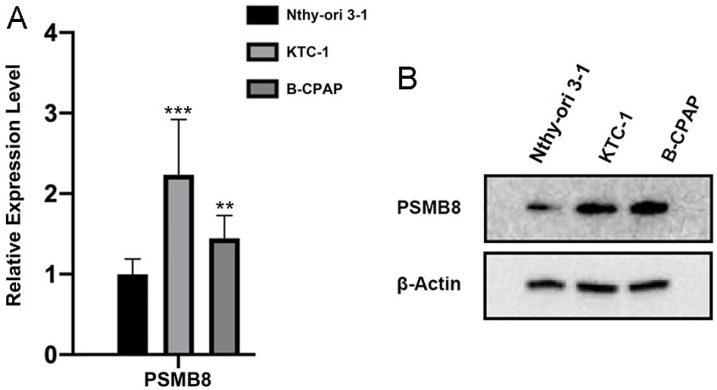
Verification of PSMB8 upregulation in THCA by *in vitro* experiments. (A) qPCR assay. **(B)** Western blot assay. PSMB8, proteasome 20S subunit beta 8; β-actin, beta-actin; THCA, thyroid carcinoma; qPCR, quantitative polymerase chain reaction.

qPCR assays revealed that the mRNA expression levels of PSMB8 were significantly higher in THCA cell lines compared to normal thyroid epithelial cell lines (Fig 9A). Similarly, western blot assays confirmed that the protein levels of PSMB8 were elevated in THCA cell lines in contrast to normal thyroid epithelial cell lines (Fig 9B).

## Discussion

Progress has been made in studying PSMB8 in relation to auto-inflammatory diseases [17,20–22,51]. Increasingly, researchers are interested in exploring the potential oncogenic molecular mechanisms of PSMB8 [23,26,33,34]. Notably, Chen *et al*. [27] conducted a comprehensive pan-cancer analysis of PSMB8 across 33 cancer types, although THCA was not their primary focus. Their analysis relevant to THCA revealed that differential expression of PSMB8 was significantly associated with OS, there is a significant positive correlation between overexpressed PSMB8 and the infiltration levels of six main immune cells, and upregulated PSMB8 is significantly associated with increased microsatellite instability (MSI) [27]. However, high-quality studies systematically elucidating the role of PSMB8 in a specific cancer type are still lacking. In this study, we aimed to explore the potential roles of PSMB8 in THCA, seeking to enrich the relevant literature and provide a basis for further elucidation and experimental validation.

The upregulation of PSMB8 was identified at both mRNA and protein levels. Significant upregulation of PSMB8 was observed in the lymph node metastasis subgroup and the extrathyroidal extension subgroup. Additionally, clinical correlation analysis, including both baseline and logistic regression analyses, indicated a correlation between higher PSMB8 expression and the lymph node metastasis as well as extrathyroidal extension. Due to the lack of relevant references, we can only preliminarily assume that PSMB8 may be involved in the biological processes of lymph node metastasis and extrathyroidal extension. High PSMB8 expression was associated with better OS and DSS for patients with THCA. In clinical subgroups, patients with higher PSMB8 expression also demonstrated favorable OS. Similar results have been observed in melanoma [52] and triple negative breast cancer [29] that patients with accumulated PSMB8 expression gained prolonged survival time. But overexpressed PSMB8 was reported to infer poor prognosis in acute myeloid leukemia [28] and glioblastoma [26]. This implied that the opposite prognostic roles of PSMB8 were largely context-dependent. Older age, higher T stage and lower PSMB8 expression were determined to be independent prognostic predictors in THCA, which was consistent with previous studies [53]. Previous studies have established gene risk signatures but not nomograms containing PSMB8 to predict prognosis risk [54,55] or metastasis risk [56] in corresponding cancer types. In our study, the nomogram constructed based on independent prognostic predictors showed robust predictive capability. Lack of external validation to the nomogram was an obvious limitation, and perhaps due to the low rate of THCA-caused death events, the C-index 0.947 (95% CI: 0.933–0.961) and AUCs (1- to 14-year) of the nomogram were extremely perfect. These may make the model misfitted to the initial dataset, capturing noise rather than the underlying pattern. Thus further researches were supposed to conduct large-cohort, multi-center, prospectively longitudinal studies to verify predictive efficacy of the nomogram. With ethnic approval, patients with THCA after surgery were involved and followed, till death or withdrawal events were recorded, thereby externally validating the nomogram and making necessary adjustment.

GO/KEGG functional enrichment analysis suggested that PSMB8 was associated with multiple immune-related signaling pathways and biological processes, especially relevant to cytokine and chemokine. Results from GSEA revealed that PSMB8 functioning was also immune-related. The top ten GSEA functional terms of PSMB8 were all immune-related. Notably, it showed that PSMB8 functioning was enriched in CTLA4 pathway and PD1 pathway. Chen *et al*. [27] got a different functional distribution pattern for PSMB8. The hallmarks for PSMB8 functioning were mainly categorized in other immune-related processes, such as antigen processing and presentation, allograft rejection, NK cell mediated cytotoxicity and IFN-γ/α response, etc. These two functional annotation patterns have indicated that PSMB8 may serve as crucial regulator in immune-related processes, especially affecting anti-tumor immunotherapy response. Actually, several previous studies have reported the modulatory role of PSMB8 for immunotherapy in multiple solid tumors. In breast cancer, Szekely *et al*. [57] found that distant metastases exhibited a significantly blunted response to immunotherapy compared to primary tumors. This was associated with a marked attenuation of multiple immune phenotypes, including decreased expression of immune checkpoints (PD1, PDL1, CTLA4), immune-related chemokines and cytokines, and antigen presentation molecules (MHC I, PSMB8, PSMB9). In melenoma, epigenetic modification of PSMB8 was strongly associated with anti-PD1/CTLA4 therapeutic efficacy. Patients with hypomethylation (thus higher expression) in the PSMB8 promoter region exhibited improved responses to immunotherapy [32]. Spatial transcriptomics analysis further supported PSMB8 as a positive predictor of immunotherapy response [58]. Similar observations were found in lung adenocarcinoma, patients with hypomethylation in PSMB8 promoter region exhibited greater activation of immune-related pathways, increased numbers of antitumor immune cells, and longer PFI following PD1/PDL1 treatment compared to those with hypermethylation in PSMB8 promoter region [30]. These results established a functional role for PSMB8 in mediating antitumor immunotherapy response, but further research was needed to elucidate the underlying mechanisms.

The proteasome complex serves as a crucial degradation terminal for cellular polyubiquitinated proteins. Foreign proteins and those damaged by cellular stress can be polyubiquitinated and targeted to the proteasome complex, thereby implicating it in the cellular stress response [59–61]. In cancer cells, common occurrences such as gene mutations and signaling pathway overloads can induce cellular stress [62]. Additionally, the tumor immune microenvironment (TIME) is often infiltrated by IFN-γ-induced lymphocytes [63]. Consequently, PSMB8, as a core subunit of the immunoproteasome, may be upregulated by cancer cells to counterbalance the accumulation of misfolded or damaged proteins produced by both cellular and immune microenvironment stresses. Our current study has primarily demonstrated potential interactions between PSMB8 and the cell-level immune network in THCA. We discovered that high PSMB8 expression significantly correlated with higher infiltration levels of 20 immune cells, including B cells, T helper cells, and cytotoxic T cells, corroborating our functional characterization results. Additionally, positive correlations between PSMB8 expression and stromal/immune scores were identified. These findings suggested a potential positive relationship between PSMB8 and immune cell enrichment in TIME, which may involve PSMB8 elevation induced by high immune cell infiltration to confront intracellular/extracellular stress. This hypothesis awaited further exploration. Importantly, we inferred that the favorable prognosis of THCA patients with higher PSMB8 expression might result from enhanced antigen presentation and cytotoxic cell-induced apoptosis of cancer cells. This inference was supported by strong associations between high PSMB8 expression and increased infiltration of three functional immune cells: aDC, cytotoxic cells, and T cells. Furthermore, in bladder cancer, PSMB8, along with seven other genes, was identified as co-expressed with CD8^+^ T cells, promoting their infiltration and correlating with improved survival in patients with higher CD8^+^ T cell infiltration [64].

Furthermore, co-expression analysis revealed that a majority of immune checkpoints were significantly positively correlated with high PSMB8 expression in THCA. Mechanically speaking, the immunoproteasome subunit PSMB8 was involved in the processing of endogenous proteins into peptides that were presented via MHC I to CD8^+^ T cells, a critical step in activating anti-tumor immunity. Elevated PSMB8 expression may enhance the presentation of thyroid cancer-associated antigens, thereby contributing to a more effective cytotoxic T cell response. These results implicated us that modulating PSMB8 activity could enhance tumor antigen processing and presentation, thereby boosting cytotoxic T cell-mediated anti-tumor immunity. This effect would be further amplified when combined with immune checkpoint blockade therapies, leading to a synergistic anti-tumor response through enhanced antigen presentation and reduced immune suppression. Besides, given that PSMB8’s strong associations with immune checkpoints and immune cells, it may serve as a promising biomarker to assess the immune activity of tumor microenvironment and predict immunotherapy response. We next checked out that PSMB8 is significantly correlated with a majority of genes from the four immune-related gene clusters. Using LASSO regression analysis, we managed to determine several immune-related genes as potential prognostic risk genes with signifcant correlation with PSMB8 in THCA. These potential risk genes may cooperate with PSMB8 in the carcinogenesis of THCA and exerting influence on prognosis.

Rising evidence has identified lncRNA-miRNA-mRNA regulating axis to be extremely activated or suppressed in cancer development and progression. Our prediction suggested two possible lncRNA-miRNA-mRNA flows to influence PSMB8 expression in THCA, they were SNHG12-miR-451a-PSMB8 and SLC25A21-AS1-miR-451a-PSMB8. PSMB8 has been reported to be a direct target of miR-451a in both PTC [34] and prostate cancer (PCa) [33] with evidence from luciferase activity reporter assay. Overexpression of miR-451a could exert inhibitive influence on PTC cells, including decreased proliferation/invasion and enhanced apoptosis, as well as in PCa cells [33,34]. Ueta *et al*. [65] observed extracellular vesicle-shuttled miR-451a is downregulated in serum from patients with gallbladder cancer, and elevation of miR-451a was able to inhibit proliferation and induce apoptosis of gallbladder cancer cells via targeting PSMB8 *in vitro*. PSMB8 has been identified as a direct target of miR-451a, implicating its involvement in childhood atopic dermatitis [66] and neuronal differentiation in mice [67]. Consequently, the ceRNA network in THCA was recommended for further investigation, highlighting upstream long non-coding RNAs (lncRNAs) for additional mechanistic research.

## Conclusion

The present study has preliminarily appraised PSMB8 as a reliable biomarker for the diagnosis and prognosis of THCA, as well as a potential target for immunotherapy. Future studies could concentrate on elucidating the intricate mechanisms linking PSMB8, TIME, and the efficacy of relevant immunotherapeutic strategies.

## Supporting information

S1 File Raw images. (DOCX)

S2 File Raw clinical data for TCGA-THCA samples. (XLSX)

## References

[1] Sung H, Ferlay J, Siegel RL, Laversanne M, Soerjomataram I, Jemal A, et al. Global Cancer Statistics 2020: GLOBOCAN estimates of incidence and mortality worldwide for 36 cancers in 185 countries. CA Cancer J Clin. 2021;71(3):209–49. doi: 10.3322/caac.21660 33538338

[2] Jung CK, Bychkov A, Kakudo K. Update from the 2022 World Health Organization classification of thyroid tumors: a standardized diagnostic approach. Endocrinol Metab (Seoul). 2022;37(5):703–18. doi: 10.3803/EnM.2022.1553 36193717 PMC9633223

[3] Prete A, Borges de Souza P, Censi S, Muzza M, Nucci N, Sponziello M. Update on fundamental mechanisms of thyroid cancer. Front Endocrinol (Lausanne). 2020;11:102. doi: 10.3389/fendo.2020.00102 32231639 PMC7082927

[4] Howlader N, Noone AM, Krapcho M. SEER cancer statistics review, 1975–2013. Bethesda: National Cancer Institute; 2016. Accessed 10 March 2024 http://seer.cancer.gov/csr/1975_2013.

[5] Asimakopoulos P, Nixon IJ, Shaha AR. Differentiated and medullary thyroid cancer: surgical management of cervical lymph nodes. Clin Oncol (R Coll Radiol). 2017;29(5):283–9. doi: 10.1016/j.clon.2017.01.001 28094086 PMC5541897

[6] Zanella AB, Scheffel RS, Weinert L, Dora JM, Maia AL. New insights into the management of differentiated thyroid carcinoma in children and adolescents (Review). Int J Oncol. 2021;58(5):13. doi: 10.3892/ijo.2021.5193 33649842

[7] Dierks C, Seufert J, Aumann K, Ruf J, Klein C, Kiefer S, et al. Combination of lenvatinib and pembrolizumab is an effective treatment option for anaplastic and poorly differentiated thyroid carcinoma. Thyroid. 2021;31(7):1076–85. doi: 10.1089/thy.2020.0322 33509020 PMC8290324

[8] Kebebew E, Clark OH. Medullary thyroid cancer. Curr Treat Options Oncol. 2000;1(4):359–67. doi: 10.1007/s11864-000-0052-7 12057161

[9] Wells SA Jr, Asa SL, Dralle H, Elisei R, Evans DB, Gagel RF, et al. Revised American Thyroid Association guidelines for the management of medullary thyroid carcinoma. Thyroid. 2015;25(6):567–610. doi: 10.1089/thy.2014.0335 25810047 PMC4490627

[10] Kloetzel PM. Generation of major histocompatibility complex class I antigens: functional interplay between proteasomes and TPPII. Nat Immunol. 2004;5(7):661–9. doi: 10.1038/ni1090 15224091

[11] Huber EM, Basler M, Schwab R, Heinemeyer W, Kirk CJ, Groettrup M, et al. Immuno- and constitutive proteasome crystal structures reveal differences in substrate and inhibitor specificity. Cell. 2012;148(4):727–38. doi: 10.1016/j.cell.2011.12.030 22341445

[12] Groettrup M, Ruppert T, Kuehn L, Seeger M, Standera S, Koszinowski U, et al. The interferon-gamma-inducible 11 S regulator (PA28) and the LMP2/LMP7 subunits govern the peptide production by the 20 S proteasome in vitro. J Biol Chem. 1995;270(40):23808–15. 7559557 10.1074/jbc.270.40.23808

[13] Toes RE, Nussbaum AK, Degermann S, Schirle M, Emmerich NP, Kraft M, et al. Discrete cleavage motifs of constitutive and immunoproteasomes revealed by quantitative analysis of cleavage products. J Exp Med 2001;194(1):1–12. 11435468 10.1084/jem.194.1.1 PMC2193442

[14] Schwarz K, van Den Broek M, Kostka S, Kraft R, Soza A, Schmidtke G, et al. Overexpression of the proteasome subunits LMP2, LMP7, and MECL-1, but not PA28 alpha/beta, enhances the presentation of an immunodominant lymphocytic choriomeningitis virus T cell epitope. J Immunol. 2000;165(2):768–78. doi: 10.4049/jimmunol.165.2.768 10878350

[15] Sijts AJ, Standera S, Toes RE, Ruppert T, Beekman NJ, van Veelen PA, et al. MHC class I antigen processing of an adenovirus CTL epitope is linked to the levels of immunoproteasomes in infected cells. J Immunol. 2000;164(9):4500–6. doi: 10.4049/jimmunol.164.9.4500 10779750

[16] Angeles A, Fung G, Luo H. Immune and non-immune functions of the immunoproteasome. Front Biosci (Landmark Ed). 2012;17(5):1904–16. doi: 10.2741/4027 22201844

[17] Agarwal AK, Xing C, DeMartino GN, Mizrachi D, Hernandez MD, Sousa AB, et al. PSMB8 encoding the β5i proteasome subunit is mutated in joint contractures, muscle atrophy, microcytic anemia, and panniculitis-induced lipodystrophy syndrome. Am J Hum Genet. 2010;87(6):866–72. doi: 10.1016/j.ajhg.2010.10.031 21129723 PMC2997366

[18] Haeussler M, Zweig AS, Tyner C, Speir ML, Rosenbloom KR, Raney BJ, et al. The UCSC Genome Browser database: 2019 update. Nucleic Acids Res. 2019;47(D1):D853–D858. 30407534 10.1093/nar/gky1095 PMC6323953

[19] Accessed 10 March 2024 http://www.genecards.org/cgi-bin/carddisp.pl?gene=PSMB8&keywords=PSMB8.

[20] Brehm A, Liu Y, Sheikh A, Marrero B, Omoyinmi E, Zhou Q, et al. Additive loss-of-function proteasome subunit mutations in CANDLE/PRAAS patients promote type I IFN production. J Clin Invest. 2015;125(11):4196–211. doi: 10.1172/JCI81260 26524591 PMC4639987

[21] Ohmura K. Nakajo-Nishimura syndrome and related proteasome-associated autoinflammatory syndromes. J Inflamm Res. 2019;12:259–65. doi: 10.2147/JIR.S194098 31576159 PMC6765212

[22] McDermott A, Jacks J, Kessler M, Emanuel PD, Gao L. Proteasome-associated autoinflammatory syndromes: advances in pathogeneses, clinical presentations, diagnosis, and management. Int J Dermatol. 2015;54(2):121–9. doi: 10.1111/ijd.12695 25521013

[23] Yang B-Y, Song J-W, Sun H-Z, Xing J-C, Yang Z-H, Wei C-Y, et al. PSMB8 regulates glioma cell migration, proliferation, and apoptosis through modulating ERK1/2 and PI3K/AKT signaling pathways. Biomed Pharm. 2018;100:205–12. doi: 10.1016/j.biopha.2018.01.170 29428669

[24] Liu R, Liu R, Guo Z, Ren J, Huang J, Luo Q, et al. shRNA‑mediated knockdown of KNTC1 inhibits non-small-cell lung cancer through regulating PSMB8. Cell Death Dis. 2022;13(8):685. doi: 10.1038/s41419-022-05140-w 35933405 PMC9357013

[25] Zhong Y, Yu F, Yang L, Wang Y, Liu L, Jia C, et al. HOXD9/miR-451a/PSMB8 axis is implicated in the regulation of cell proliferation and metastasis via PI3K/AKT signaling pathway in human anaplastic thyroid carcinoma. J Transl Med. 2023;21(1):817. doi: 10.1186/s12967-023-04538-0 37974228 PMC10652604

[26] Chang H-H, Cheng Y-C, Tsai W-C, Chen Y. PSMB8 inhibition decreases tumor angiogenesis in glioblastoma through vascular endothelial growth factor A reduction. Cancer Sci. 2020;111(11):4142–53. doi: 10.1111/cas.14625 32816328 PMC7648028

[27] Chen D, Jin C, Dong X, Wen J, Xia E, Wang Q, et al. Pan-cancer analysis of the prognostic and immunological role of PSMB8. Sci Rep. 2021;11(1):20492. doi: 10.1038/s41598-021-99724-9 34650125 PMC8516870

[28] Zhang Y, Xue S, Hao Q, Liu F, Huang W, Wang J. Galectin-9 and PSMB8 overexpression predict unfavorable prognosis in patients with AML. J Cancer. 2021;12(14):4257–63. doi: 10.7150/jca.53686 34093826 PMC8176406

[29] Geoffroy K, Araripe Saraiva B, Viens M, Béland D, Bourgeois-Daigneault M-C. Increased expression of the immunoproteasome subunits PSMB8 and PSMB9 by cancer cells correlate with better outcomes for triple-negative breast cancers. Sci Rep. 2023;13(1):2129. doi: 10.1038/s41598-023-28940-2 36746983 PMC9902398

[30] Xie T, Fan G, Huang L, Lou N, Han X, Xing P, et al. Analysis on methylation and expression of PSMB8 and its correlation with immunity and immunotherapy in lung adenocarcinoma. Epigenomics. 2022;14(22):1427–48. doi: 10.2217/epi-2022-0282 36683462

[31] Ha YJ, Tak KH, Kim CW, Roh SA, Choi EK, Cho DH, et al. PSMB8 as a candidate marker of responsiveness to preoperative radiation therapy in rectal cancer patients. Int J Radiat Oncol Biol Phys. 2017;98(5):1164–73. doi: 10.1016/j.ijrobp.2017.03.023 28721901

[32] Newell F, Pires da Silva I, Johansson PA, Menzies AM, Wilmott JS, Addala V, et al. Multiomic profiling of checkpoint inhibitor-treated melanoma: Identifying predictors of response and resistance, and markers of biological discordance. Cancer Cell. 2022;40(1):88-102.e7. doi: 10.1016/j.ccell.2021.11.012 34951955

[33] Liu Y, Yang H-Z, Jiang Y-J, Xu L-Q. miR-451a is downregulated and targets PSMB8 in prostate cancer. Kaohsiung J Med Sci. 2020;36(7):494–500. doi: 10.1002/kjm2.12196 32128987 PMC11896265

[34] Fan X, Zhao Y. miR-451a inhibits cancer growth, epithelial-mesenchymal transition and induces apoptosis in papillary thyroid cancer by targeting PSMB8. J Cell Mol Med. 2019;23(12):8067–75. doi: 10.1111/jcmm.14673 31559672 PMC6850967

[35] Szklarczyk D, Morris JH, Cook H, Kuhn M, Wyder S, Simonovic M, et al. The STRING database in 2017: quality-controlled protein-protein association networks, made broadly accessible. Nucleic Acids Res. 2017;45(D1):D362–8. doi: 10.1093/nar/gkw937 27924014 PMC5210637

[36] Love MI, Huber W, Anders S. Moderated estimation of fold change and dispersion for RNA-seq data with DESeq2. Genome Biol. 2014;15(12):550. doi: 10.1186/s13059-014-0550-8 25516281 PMC4302049

[37] Yu G, Wang L-G, Han Y, He Q-Y. clusterProfiler: an R package for comparing biological themes among gene clusters. OMICS. 2012;16(5):284–7. doi: 10.1089/omi.2011.0118 22455463 PMC3339379

[38] Subramanian A, Tamayo P, Mootha VK, Mukherjee S, Ebert BL, Gillette MA, et al. Gene set enrichment analysis: a knowledge-based approach for interpreting genome-wide expression profiles. Proc Natl Acad Sci U S A. 2005;102(43):15545–50. doi: 10.1073/pnas.0506580102 16199517 PMC1239896

[39] Hänzelmann S, Castelo R, Guinney J. GSVA: gene set variation analysis for microarray and RNA-seq data. BMC Bioinform. 2013;14:7. doi: 10.1186/1471-2105-14-7 23323831 PMC3618321

[40] Bindea G, Mlecnik B, Tosolini M, Kirilovsky A, Waldner M, Obenauf AC, et al. Spatiotemporal dynamics of intratumoral immune cells reveal the immune landscape in human cancer. Immunity. 2013;39(4):782–95. doi: 10.1016/j.immuni.2013.10.003 24138885

[41] Yoshihara K, Shahmoradgoli M, Martínez E, Vegesna R, Kim H, Torres-Garcia W, et al. Inferring tumour purity and stromal and immune cell admixture from expression data. Nat Commun. 2013;4:2612. doi: 10.1038/ncomms3612 24113773 PMC3826632

[42] Chen Y-J, Liao W-X, Huang S-Z, Yu Y-F, Wen J-Y, Chen J, et al. Prognostic and immunological role of CD36: a pan-cancer analysis. J Cancer. 2021;12(16):4762–73. doi: 10.7150/jca.50502 34234847 PMC8247371

[43] Cheng X, Wang X, Nie K, Cheng L, Zhang Z, Hu Y, et al. Systematic pan-cancer analysis identifies TREM2 as an immunological and prognostic biomarker. Front Immunol. 2021;12:646523. 33679809 10.3389/fimmu.2021.646523 PMC7925850

[44] Wei W-J, Sun Z-K, Shen C-T, Song H-J, Zhang X-Y, Qiu Z-L, et al. Obatoclax and LY3009120 efficiently overcome vemurafenib resistance in differentiated thyroid cancer. Theranostics. 2017;7(4):987–1001. doi: 10.7150/thno.17322 28382170 PMC5381260

[45] You M-H, Lee WK, Jin M, Song DE, Cheng S-Y, Kim TY, et al. Death-associated protein kinase 1 inhibits progression of thyroid cancer by regulating stem cell markers. Cells. 2021;10(11):2994. doi: 10.3390/cells10112994 34831219 PMC8616132

[46] Kim HJ, Nguyen QK, Jung S-N, Lim MA, Oh C, Piao Y, et al. Mitochondrial ribosomal protein L14 promotes cell growth and invasion by modulating reactive oxygen species in thyroid cancer. Clin Exp Otorhinolaryngol. 2023;16(2):184–97. doi: 10.21053/ceo.2022.01760 36822197 PMC10208851

[47] Li Y, Zhou X, Zhang Q, Chen E, Sun Y, Ye D, et al. Lipase member H is a downstream molecular target of hypoxia inducible factor-1α and promotes papillary thyroid carcinoma cell migration in BCPAP and KTC-1 cell lines. Cancer Manag Res. 2019;11:931–41. doi: 10.2147/CMAR.S183355 30774423 PMC6349079

[48] Wang Z, Yang B, Zhang M, Guo W, Wu Z, Wang Y, et al. lncRNA epigenetic landscape analysis identifies EPIC1 as an oncogenic lncRNA that interacts with MYC and promotes cell-cycle progression in cancer. Cancer Cell. 2018;33(4):706-720.e9. doi: 10.1016/j.ccell.2018.03.006 29622465 PMC6143179

[49] Lu X, Zhang C, Zhu L, Wang S, Zeng L, Zhong W, et al. TBL2 promotes tumorigenesis via PRMT5/WDR77-mediated AKT activation in breast cancer. Adv Sci (Weinh). 2024;11(47):e2400160. doi: 10.1002/advs.202400160 39499734 PMC11653647

[50] Xu J, Zhang Z, Qian M, Wang S, Qiu W, Chen Z, et al. Cullin-7 (CUL7) is overexpressed in glioma cells and promotes tumorigenesis via NF-κB activation. J Exp Clin Cancer Res. 2020;39(1):59. 32252802 10.1186/s13046-020-01553-7 PMC7132976

[51] de Jesus AA, Hou Y, Brooks S, Malle L, Biancotto A, Huang Y, et al. Distinct interferon signatures and cytokine patterns define additional systemic autoinflammatory diseases. J Clin Invest. 2020;130(4):1669–82. doi: 10.1172/JCI129301 31874111 PMC7108905

[52] Kalaora S, Lee JS, Barnea E, Levy R, Greenberg P, Alon M, et al. Immunoproteasome expression is associated with better prognosis and response to checkpoint therapies in melanoma. Nat Commun. 2020;11(1):896. doi: 10.1038/s41467-020-14639-9 32060274 PMC7021791

[53] Wang J, Zhanghuang C, Jin L, Zhang Z, Tan X, Mi T, et al. Development and validation of a nomogram to predict cancer-specific survival in elderly patients with papillary thyroid carcinoma: a population-based study. BMC Geriatr. 2022;22(1):736. doi: 10.1186/s12877-022-03430-8 36076163 PMC9454205

[54] Tian W, Chen K, Yan G, Han X, Liu Y, Zhang Q, et al. A novel prognostic tool for glioma based on enhancer RNA-regulated immune genes. Front Cell Dev Biol. 2022;9:798445. doi: 10.3389/fcell.2021.798445 35127714 PMC8811171

[55] He T, Huang L, Li J, Wang P, Zhang Z. Potential prognostic immune biomarkers of overall survival in ovarian cancer through comprehensive bioinformatics analysis: a novel artificial intelligence survival prediction system. Front Med (Lausanne). 2021;8:587496. doi: 10.3389/fmed.2021.587496 34109184 PMC8180546

[56] Fernandez-Retana J, Zamudio-Meza H, Rodriguez-Morales M, Pedroza-Torres A, Isla-Ortiz D, Herrera L, et al. Gene signature based on degradome-related genes can predict distal metastasis in cervical cancer patients. Tumour Biol. 2017;39(6):1010428317711895. 28639897 10.1177/1010428317711895

[57] Szekely B, Bossuyt V, Li X, Wali VB, Patwardhan GA, Frederick C, et al. Immunological differences between primary and metastatic breast cancer. Ann Oncol. 2018;29(11):2232–2239. 30203045 10.1093/annonc/mdy399

[58] Aung TN, Warrell J, Martinez-Morilla S, Gavrielatou N, Vathiotis I, Yaghoobi V, et al. Spatially informed gene signatures for response to immunotherapy in melanoma. Clin Cancer Res. 2024;30(16):3520–32. doi: 10.1158/1078-0432.CCR-23-3932 38837895 PMC11326985

[59] Flick K, Kaiser P. Protein degradation and the stress response. Semin Cell Dev Biol. 2012;23(5):515–22. doi: 10.1016/j.semcdb.2012.01.019 22414377 PMC3376211

[60] Granados DP, Tanguay P-L, Hardy M-P, Caron E, de Verteuil D, Meloche S, et al. ER stress affects processing of MHC class I-associated peptides. BMC Immunol. 2009;10:10. doi: 10.1186/1471-2172-10-10 19220912 PMC2657905

[61] Meiners S, Eickelberg O. What shall we do with the damaged proteins in lung disease? Ask the proteasome!. Eur Respir J. 2012;40(5):1260–8. doi: 10.1183/09031936.00208511 22441749

[62] Luo J, Solimini NL, Elledge SJ. Principles of cancer therapy: oncogene and non-oncogene addiction. Cell. 2009;138(4):807. doi: 10.1016/j.cell.2009.08.006 PMC2894612 19269363

[63] Mlecnik B, Bindea G, Kirilovsky A, Angell HK, Obenauf AC, Tosolini M, et al. The tumor microenvironment and immunoscore are critical determinants of dissemination to distant metastasis. Sci Transl Med. 2016;8(327):327ra26. 10.1126/scitranslmed.aad6352 26912905

[64] Wang Y, Yan K, Lin J, Liu Y, Wang J, Li X, et al. CD8 T cell co-expressed genes correlate with clinical phenotype and microenvironments of urothelial cancer. Front Oncol. 2020;10:553399. 33330025 10.3389/fonc.2020.553399 PMC7713665

[65] Ueta E, Tsutsumi K, Kato H, Matsushita H, Shiraha H, Fujii M, et al. Extracellular vesicle-shuttled miRNAs as a diagnostic and prognostic biomarker and their potential roles in gallbladder cancer patients. Sci Rep. 2021;11(1):12298. doi: 10.1038/s41598-021-91804-0 34112884 PMC8192895

[66] Nousbeck J, McAleer MA, Hurault G, Kenny E, Harte K, Kezic S, et al. MicroRNA analysis of childhood atopic dermatitis reveals a role for miR-451a. Br J Dermatol. 2021;184(3):514–23. doi: 10.1111/bjd.19254 32478410

[67] Trattnig C, Üçal M, Tam-Amersdorfer C, Bucko A, Zefferer U, Grünbacher G, et al. MicroRNA-451a overexpression induces accelerated neuronal differentiation of Ntera2/D1 cells and ablation affects neurogenesis in microRNA-451a-/- mice. PLoS One. 2018;13(11):e0207575. doi: 10.1371/journal.pone.0207575 30462722 PMC6248975

