# Correction: Multifaceted investigations of PSMB8 provides insights into prognostic prediction and immunological target in thyroid carcinoma

**DOI:** 10.1371/journal.pone.0327913

**Published:** 2025-07-09

**Authors:** The PLOS One Staff

The fifth author, Yan Chen, should have been noted as corresponding author. Yan Chen’s email address is: yanchen@xjmu.edu.cn.

The publisher apologizes for the error.

## References

[1] Luo Y, Ye Y, Saibaidoula Y, Zhang Y, Chen Y. Multifaceted investigations of PSMB8 provides insights into prognostic prediction and immunological target in thyroid carcinoma. PLoS One. 2025;20(5):e0323013. doi: 10.1371/journal.pone.0323013 40334200 PMC12058196

