# Supplementary material for: Multifaceted investigations of PSMB8 provides insights into prognostic prediction and immunological target in thyroid carcinoma
Source: PLoS One. 2025 May 7;20(5):e0323013. doi: 10.1371/journal.pone.0323013 (PMC12058196; doi:10.1371/journal.pone.0323013)
Supplement: S1 File — (DOCX) [file pone.0323013.s001.docx]

**Gradient for biomarker molecular mass:**


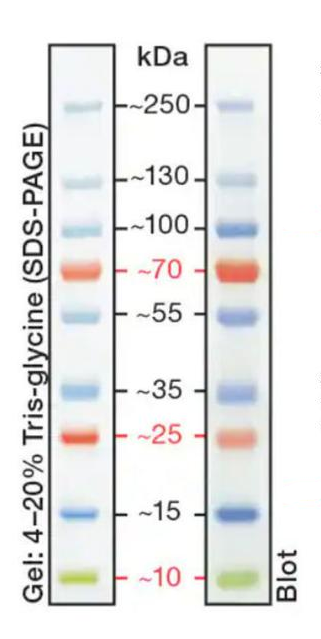


**Raw bolt images for PSMB8 (30 kDa):**


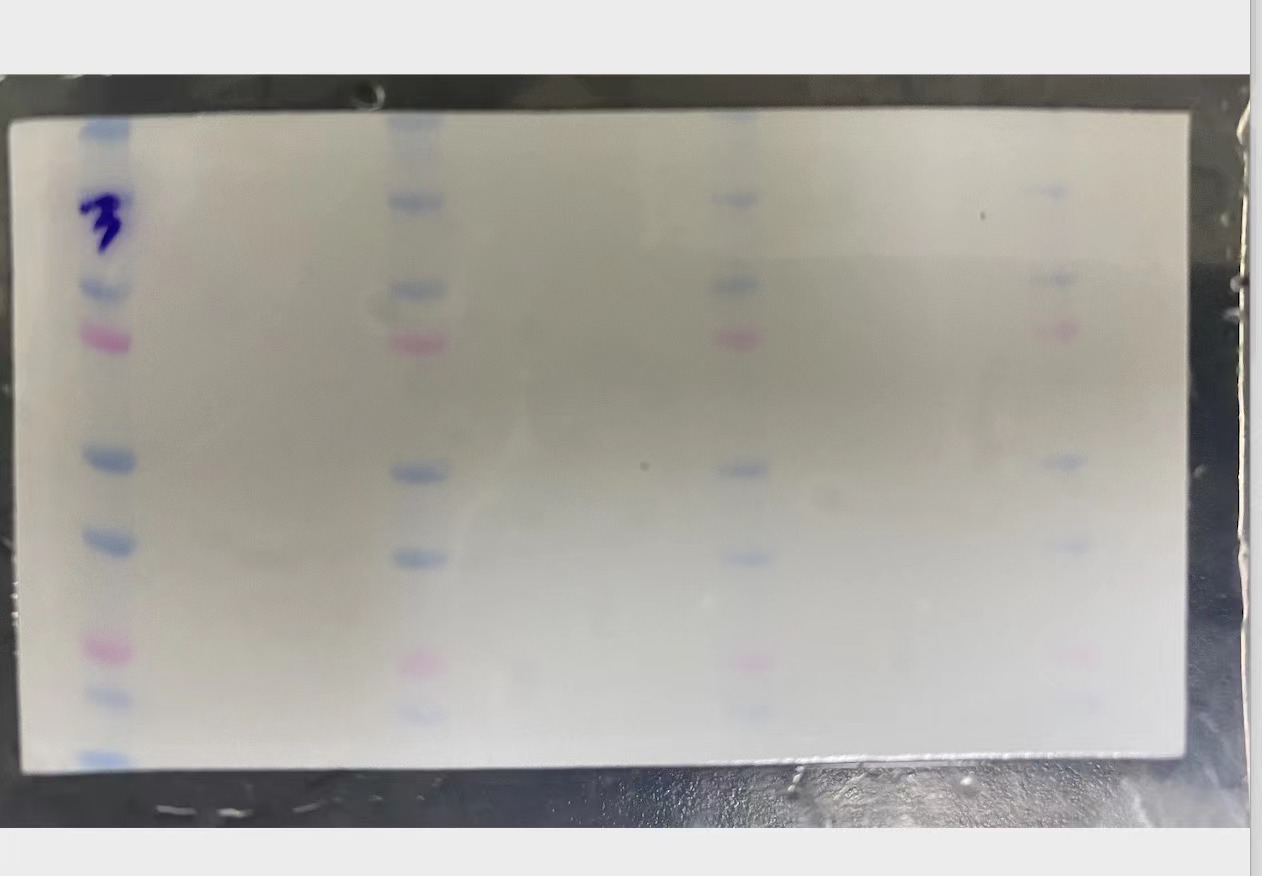

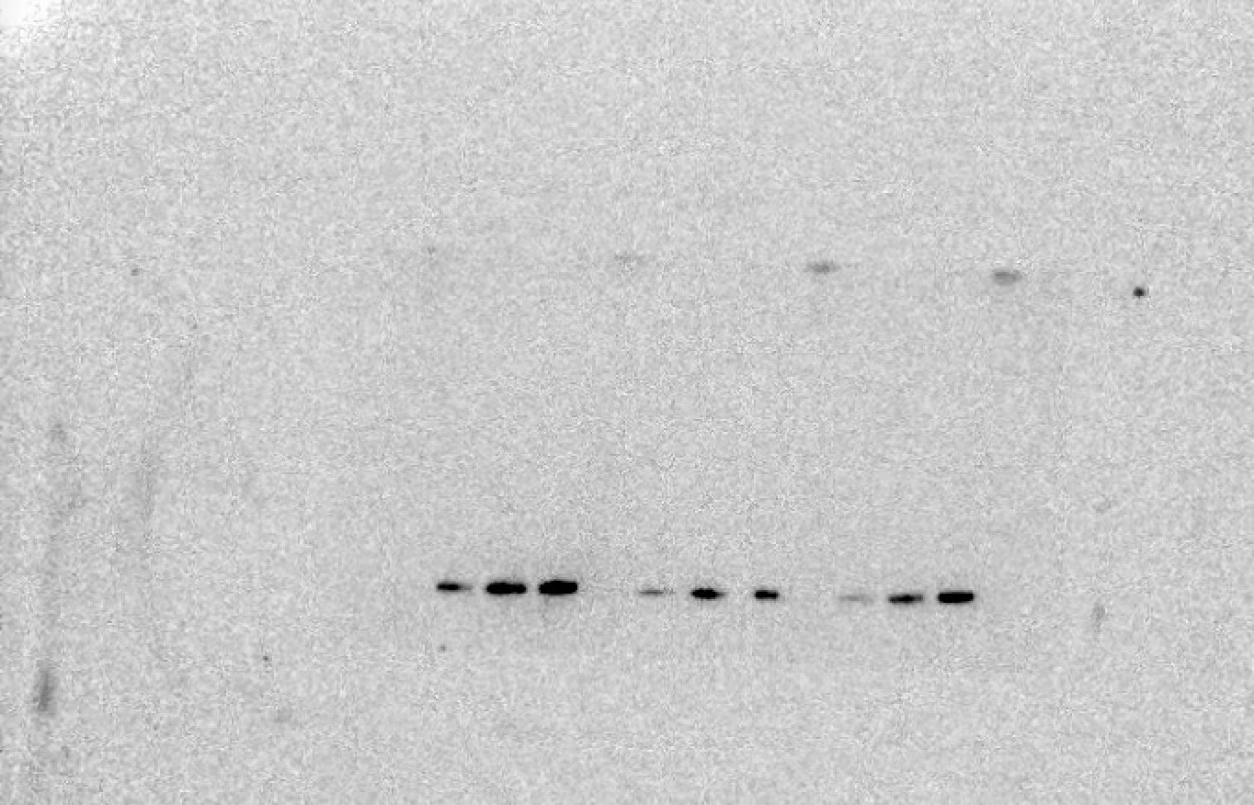


**×**

**×**

**×**

**×**

**×**

**×**

30 kDa

30 kDa

10 kDa

15 kDa

25 kDa

35 kDa

55 kDa

70 kDa

100 kDa

130 kDa

250 kDa

**① ② ③ ④ ⑤ ⑥ ⑦ ⑧ ⑨**

-Loading order: Normal thyroid epithelial cell line Nthy-ori 3-1, thyroid cancer cell line KTC-1, thyroid cancer cell line B-CPAP.

-Experimental samples: ①Normal thyroid epithelial cell line Nthy-ori 3-1, ②Thyroid cancer cell line KTC-1, ③Thyroid cancer cell line B-CPAP, ④⑤⑥Replication once, ⑦⑧⑨Replication twice.

-Method used to capture the image: The blots were enhanced using a chemiluminescent kit (Vazyme, Nanjing, China). All images were captured by the Bio-Rad Imaging System.

-Which figure panel was generated from this original image: Figure 9B.

**Raw bolt images for β-actin (41 kDa):**


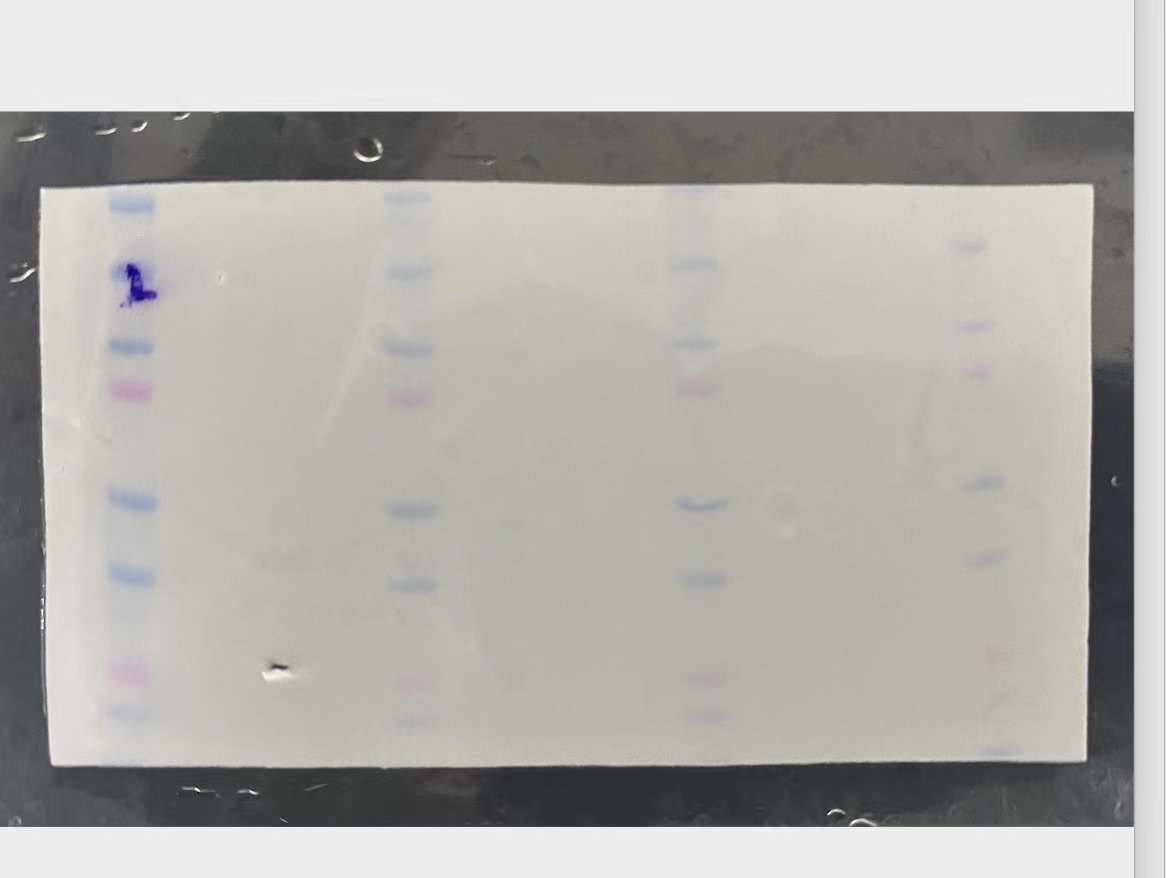


41 kDa

15 kDa

25 kDa

10 kDa

55 kDa

35 kDa

250 kDa

130 kDa

100 kDa

70 kDa


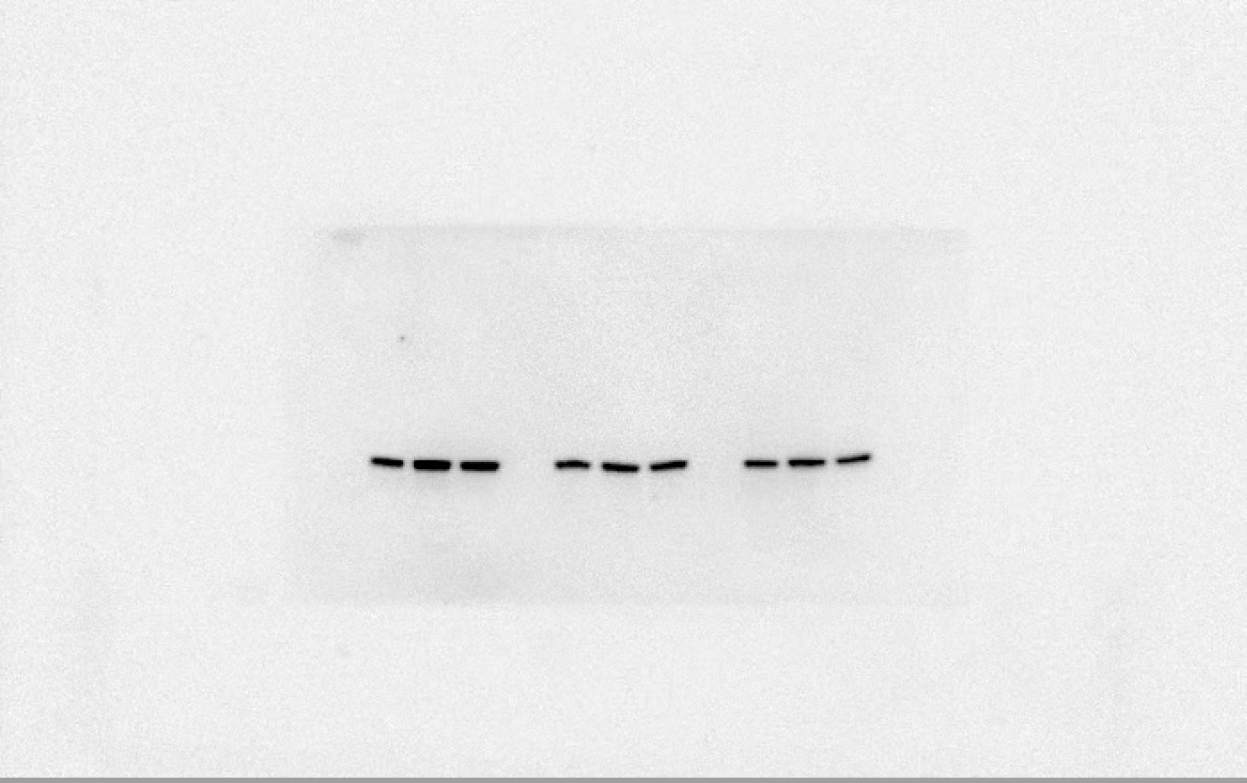


**×**

**×**

**×**

**×**

**×**

**×**

41 kDa

**① ② ③ ④ ⑤ ⑥ ⑦ ⑧ ⑨**

-Loading order: Normal thyroid epithelial cell line Nthy-ori 3-1, thyroid cancer cell line KTC-1, thyroid cancer cell line B-CPAP.

-Experimental samples: ①Normal thyroid epithelial cell line Nthy-ori 3-1, ②Thyroid cancer cell line KTC-1, ③Thyroid cancer cell line B-CPAP, ④⑤⑥Replication once, ⑦⑧⑨Replication twice.

-Method used to capture the image: The blots were enhanced using a chemiluminescent kit (Vazyme, Nanjing, China). All images were captured by the Bio-Rad Imaging System.

-Which figure panel was generated from this original image: Figure 9B.
